# Baseline proteinuria level is associated with prognosis in idiopathic membranous nephropathy

**DOI:** 10.1080/0886022X.2019.1605294

**Published:** 2019-05-06

**Authors:** Xinxin Chen, Yu Chen, Xiaokai Ding, Ying Zhou, Yinqiu Lv, Duo Li, Bo Chen, Tianxin Chen, Chaosheng Chen

**Affiliations:** a Department of Nephrology, The First Affiliated Hospital of Wenzhou Medical University, Wenzhou, China;; b Department of Nephrology, Zhejiang Chinese Medical University Affiliated Wenzhou Hospital of Traditional Chinese Medicine, Wenzhou, China

**Keywords:** Idiopathic membranous nephropathy, proteinuria, prognosis, end-stage renal disease, remission

## Abstract

**Objectives:** This study aimed to investigate the unique prognostic, clinical, and renal histopathological characteristics of patients with idiopathic membranous nephropathy (IMN) with different levels of proteinuria.

**Methods:** This retrospective observational study included 190 IMN patients with low levels of proteinuria (low group), 193 IMN patients with medium levels of proteinuria (medium group), and 123 IMN patients with high levels of proteinuria (high group) treated between September 2006 and November 2015. Prognostic and baseline clinical and histopathological data were compared among the three groups. Poor prognostic events included the occurrence of a persistent 50% reduction in estimated glomerular filtration rate (eGFR), end-stage renal disease, or all-cause mortality.

**Results:** The severity of clinical symptoms and laboratory indices, such as blood pressure; extent of edema and hematuria; levels of fibrinogen, immunoglobulin (Ig)-G, complement (C)-4, total protein, albumin (ALB), and serum creatinine (SCr); and eGFR increased with increasing proteinuria (all *p*< .001). Based on renal histopathology, the extent of segmental sclerosis and balloon adhesion and renal interstitial lesion stage also increased in severity with increasing proteinuria (all *p*< .001). The Kaplan–Meier analysis showed that compared with patients with low and medium levels of proteinuria, patients with high levels of proteinuria had significantly lower cumulative poor event-free renal survival rates (*p*= .0039).

**Conclusions:** Baseline proteinuria level is indicative of prognosis in IMN patients; the greater the extent of proteinuria is, the worse the prognosis.

Membranous nephropathy (MN), an autoimmune glomerular disease [1], is one of the most common causes of nephrotic syndrome in adults [2–5], accounting for up to one-third of biopsy diagnoses. MN is defined at the histopathologic level by the presence of immune complexes on the extracapillary side of the glomerular basement membrane. Approximately, 75% of MN cases are idiopathic or primary membranous nephropathy (IMN) [3], with a peak incidence during the fourth and fifth decades of life and a male to female ratio of 2–3:1 [6,7]. The remaining proportion of MN is associated with a variety of conditions that lead to secondary MN [8]. The exact pathogenesis of MN is still unclear. Moreover, the course of MN is quite variable, with approximately one-third of patients undergoing spontaneous remission of proteinuria, another one-third experiencing persistent proteinuria, and the remaining one-third progressing to end-stage renal failure [9–13].

Recent clinical studies demonstrated that persistent nephrotic-range proteinuria and elevated creatinine levels are associated with poor outcomes [14,15]. Therefore, a question remains regarding whether baseline proteinuria is less important than persistent heavy proteinuria. In this study, we investigated baseline proteinuria levels to study whether the level of proteinuria at the time of renal biopsy is associated with long-term prognosis.

## Materials and methods

### Ethics statement

The study was approved by The First Affiliated Hospital of Wenzhou Medical University (No.: 2016151).

### Patient selection

**Figure 1.**
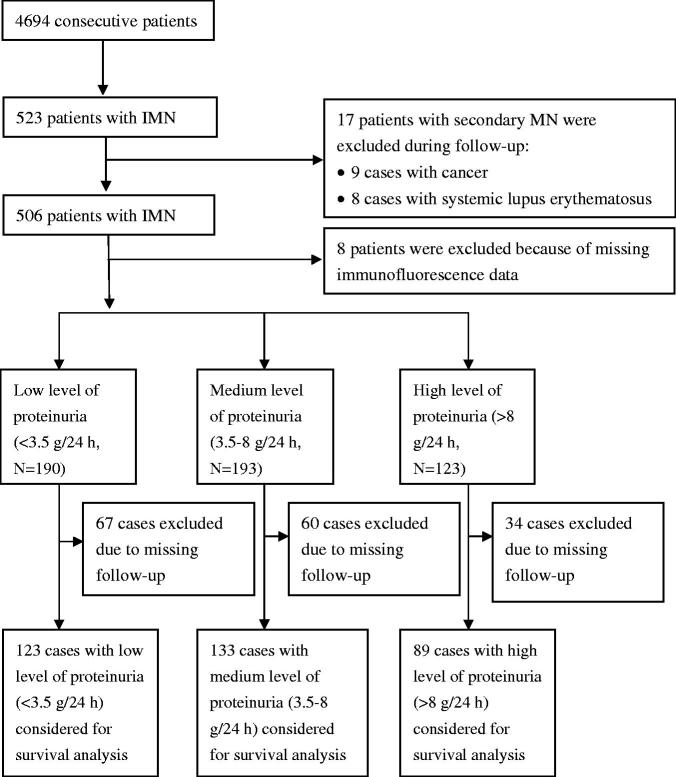
Patient selection flowchart.

In total, 506 patients with IMN were identified between 11 September 2006, and 25 November 2015, in The First Affiliated Hospital of Wenzhou Medical University. The diagnostic criteria for IMN and exclusion criteria were same as those used in our previous study [16]. The flowchart detailing the patient selection process is displayed in Figure 1. The baseline date for the analysis was the same as the date of biopsy, which also marked the start of the follow-up period. The baseline clinical data and pathological indicators were collected as in our previous study [16].

Urinary protein levels were measured in 24-h urine samples. Nephrotic syndrome was defined as proteinuria ≥ 3.5 g/24 h and hypoalbuminemia (<30 g/L). A sub-nephrotic state was defined as proteinuria > 0.5 g/24 h and <3.5 g/24 h. Urine protein levels were graded from 1 to 3, which corresponded with <3.5 g, 3.5–8 g, and >8 g or low, medium and high levels of proteinuria, respectively. Urinary sediment, estimated based on proteinuria and microscopic hematuria, was graded on a scale from 0 to 4.

Kidney biopsies were evaluated by light microscopy, immunofluorescence, and electron microscopy. Sections for light microscopy were prepared from formalin-fixed paraffin-embedded tissues and subjected to H&E, PAS, PASM, and Masson staining. The biopsies were evaluated in detail for the following features: inflammatory cell infiltration, interstitial fibrosis, tubular atrophy, and renal interstitial lesion stage graded in a semiquantitative manner from 0 to 3 (0: 0–4% in area, 1: 5–24% in area, 2: 25–49% in area, or 3: 50% or greater in area); the other pathological indicators included the presence or absence of segmental sclerosis and balloon adhesion. Immunofluorescence studies were performed to diagnose IMN. The expression of immunoglobulin (Ig)G, IgA, IgM, C3, C4, C1q, and fibrinogen detected by immunofluorescence was classified from 0 to 4, representative of the intensity of immunofluorescence-positive deposits. Electron microscopic studies were performed in all cases of IMN. Ultrathin sections from Epon-embedded tissue samples were sequentially fixed in 2.5% glutaraldehyde and 1% osmium tetroxide before being stained with uranyl acetate and lead citrate, and then the sections were examined with a Hitachi H7500 electron microscope (Hitachi, Tokyo, Japan). MN was staged from 1 to 4 by electron microscopy based on the ultrastructural criteria of Ehrenreich and Churg.

### Outcome parameters

Complete remission (CR) and partial remission (PR) were the outcomes and prognostic parameters that were recorded and followed, as in our previous study [16].

### Statistical analysis

All data are presented as the means ± standard deviations (SDs). Analysis of variance, the chi-square test, the Kaplan–Meier (K–M) method, the log-rank test, and univariate and multivariate Cox regression analyses were used as in our previous study [16]. MedCalc version 15.6 (Ostend, Belgium) and SPSS version 23 (SPSS Inc., Chicago, IL) were used for the statistical analyses. All *p* values were two-sided, and *p*< .05 was considered statistically significant.

## Results

Of the 506 patients with IMN, 190 had low levels of proteinuria (low group), 193 had medium levels of proteinuria (medium group), and 123 had high levels of proteinuria (high group). After 6 months, 123 patients in the low group, 133 patients in the medium group and 89 patients in the high group were included in the outcome and prognostic analyses after the exclusion of those without complete follow-up data. The patient selection flowchart is displayed in Figure 1.

### Comparison of baseline clinical characteristics, histopathological examination, and immunofluorescence staining of renal biopsies among groups with low, medium, or high level of proteinuria

The low, medium, and high groups differed in terms of sex, history of hypertension, presence of edema and hematuria, levels of fibrinogen, IgG, C4, total protein (TP), ALB, and serum creatinine (SCr), and estimated glomerular filtration rate (eGFR) evaluated by the MDRD equation. Male patients were more likely to have higher levels of proteinuria than female patients, representing 40.5%, 53.9%, and 74.8% of patients in the low, medium, and high groups, respectively. Patients in the high group had higher systolic and diastolic blood pressure (137.02 ± 19.48 mmHg and 82.57 ± 13.42 mmHg, respectively), were more prone to have edema (93.5%) and hematuria (55.3%), had higher levels of fibrinogen (5.90 ± 3.19 g/L), C4 (0.25 ± 0.08 g/L), and SCr (77.98 ± 26.66 µmol/L), and had lower levels of IgG (5.43 ± 2.56 g/L), TP (43.23 ± 7.11 g/L), and ALB (19.14 ± 4.46 g/L), and lower eGFR evaluated by the MDRD Equation (104.02 ± 35.76 mL/min/1.73 m^2^) than did patients in the low and medium groups (all *p* < .001). However, age at disease onset, infection status, presence of embolism, and levels of IgA, IgM, C3, and blood urea nitrogen (BUN) were comparable among the three groups (Table 1).

**Table 1.** Baseline clinical characteristics.

	Low level of proteinuria (<3.5 g/24 h, *N* = 190)	Medium level of proteinuria (<3.5–8 g/24 h, *N* = 193)	High level of proteinuria (>8 g/24 h, *N* = 123)	*p* Value
Age at disease onset (years)	49.04 ± 14.11	51.16 ± 15.40	48.53 ± 16.45	.241
Men,*n* (%)	77 (40.5)	104 (53.9)	92 (74.8)	**<.001**
Systolic pressure (mmHg)	128.03 ± 20.36	132.53 ± 19.90	137.02 ± 19.48	**.001**
Diastolic pressure (mmHg)	76.68 ± 12.66	77.91 ± 13.24	82.57 ± 13.42	**<.001**
Edema,*n* (%)	128 (67.4)	168 (87.0)	115 (93.5)	**<.001**
Microscopic hematuria,*n* (%)	64 (33.7)	78 (40.4)	68 (55.3)	**.001**
Infection,*n* (%)	23 (12.1)	40 (20.7)	21 (17.1)	.076
Embolism,*n* (%)	9 (4.7)	5 (2.6)	5 (4.1)	.519
Fibrinogen (g/L)	4.28 ± 2.95	4.71 ± 1.12	5.90 ± 3.19	**<.001**
Immunoglobulin G (g/L)	8.31 ± 2.78	6.70 ± 2.42	5.43 ± 2.56	**<.001**
Immunoglobulin A (g/L)	2.26 ± 0.99	2.21 ± 0.86	2.07 ± 0.88	.205
Immunoglobulin M (g/L)	1.37 ± 0.83	1.41 ± 0.72	1.32 ± 0.63	.576
Complement 3 (g/L)	1.00 ± 0.21	1.02 ± 0.21	1.02 ± 0.22	.521
Complement 4 (g/L)	0.22 ± 0.07	0.24 ± 0.07	0.25 ± 0.08	**.001**
Total protein (g/L)	55.04 ± 7.84	48.99 ± 7.85	43.23 ± 7.11	**<.001**
Albumin (g/L)	28.85 ± 6.05	23.74 ± 5.34	19.14 ± 4.46	**<.001**
Blood urea nitrogen (mmol/L)	4.95 ± 1.72	5.21 ± 2.22	5.43 ± 2.84	.177
Serum creatinine (μmol/L)	61.61 ± 18.10	66.72 ± 22.86	77.98 ± 26.66	**<.001**
eGFR (mL/min/1.73 m^2^)	117.32 ± 30.65	111.59 ± 28.73	104.02 ± 35.76	**.001**

eGFR: estimated glomerular filtration rate based on the abbreviated MDRD equation.

Data are presented as the mean ± SD or number of cases (% of total cases). Analysis of variance or the chi-square test was used to compare the three groups. *p* < .05 was shown in bold.

Regarding pathological aspects, although pathological changes were comparable among the low, medium, and high groups, the extent of segmental sclerosis and balloon adhesion and renal interstitial lesion stage increased in severity with increasing proteinuria, especially in the high group with values of 19.5% (*p*= .008), 20.3% (*p*= .010), and 0.82 ± 0.64 (*p*= .001), respectively. In addition, based on immunofluorescence, the signal for C3 increased (1.82 ± 0.87, *p*= .004) and the for fibrinogen decreased (0.18 ± 0.53, *p*= .014) with increasing proteinuria. Nevertheless, the fluorescence signals for IgG, IgA, IgM, C4, and C1q were comparable among the three groups (Table 2).

**Table 2.** Baseline histopathological characteristics and immunofluorescence staining of renal biopsies.

	Low level of proteinuria (<3.5 g/24 h,*N*=190)	Medium level of proteinuria (<3.5–8 g/24 h,*N*=193)	High level of proteinuria (>8 g/24 h,*N*=123)	*p* Value
Pathological stage	1.61 ± 0.61	1.72 ± 0.62	1.76 ± 0.53	.069
Segmental sclerosis,*n* (%)	16 (8.4)	34 (17.6)	24 (19.5)	**.008**
Balloon adhesion	17 (8.9)	34 (17.6)	25 (20.3)	**.010**
Renal interstitial lesion stage	0.57 ± 0.57	0.73 ± 0.54	0.82 ± 0.64	**.001**
Immunofluorescence staining (staining intensity score)				
IgG	2.76 ± 0.57	2.79 ± 0.51	2.87 ± 0.46	.202
IgA	0.39 ± 0.83	0.35 ± 0.86	0.33 ± 0.77	.774
IgM	0.45 ± 0.77	0.35 ± 0.68	0.28 ± 0.61	.126
C3	1.48 ± 0.88	1.64 ± 0.86	1.82 ± 0.87	**.004**
C4	0.08 ± 0.39	0.13 ± 0.37	0.13 ± 0.42	.635
C1q	0.33 ± 0.63	0.31 ± 0.59	0.38 ± 0.62	.622
Fibrinogen	0.39 ± 0.73	0.25 ± 0.61	0.18 ± 0.53	**.014**

Data are presented as the mean ± SD of severity score or staining intensity or as the number of positive cases (% of total cases). Analysis of variance or the chi-square test was used to compare the three groups. *p* < .05 was shown in bold.

### Patients with high levels of proteinuria had significantly lower cumulative poor event-free renal survival rates than did patients with low or medium levels of proteinuria

The patients’ choices regarding treatment were analyzed, as in our previous study [16]. Compared with patients in the medium and high groups, patients in the low group were more likely to choose RAS receptor inhibitors (26.3% and 12.4% versus 58.5%, respectively, *p*< .001). However, compared with patients in the low and medium groups, patients in the high group were more likely to choose GC + CTX and CNI (tacrolimus or cyclosporine) (GC + CTX, 11.4% and 16.5% versus 39.3%, respectively, *p*< .001 and CNI, 16.3% and 43.6% versus 41.6%, respectively *p*< .001) (Table 3).

**Table 3.** Different treatments with at least 6 months of follow-up in patients with low, medium or high levels of proteinuria.

Treatment	Low level of proteinuria (<3.5 g/24 h, *N* = 123)	Medium level of proteinuria (<3.5–8 g/24 h, *N* = 133)	High level of proteinuria (>8 g/24 h, *N* = 89)	*p* Value
Without treatment	6 (4.9%)	7 (5.3%)	3 (3.4%)	.785
RAS receptor inhibitors	72 (58.5%)	35 (26.3%)	11 (12.4%)	**<.001**
GC + CTX	14 (11.4%)	22 (16.5%)	35 (39.3%)	**<.001**
CNI	20 (16.3%)	58 (43.6%)	37 (41.6%)	**<.001**

(1) Without treatment: general treatment without RAS inhibitors, GC or immunosuppressive agents; (2) RAS receptor inhibitors: double dose of ACEis or ARBs given orally per day; (3) GC + CTX: full oral dose (1 mg/kg/d) of GC per day and i.v. 1 g/month CTX until an accumulated dose of 6–8 g for 6 months; (4) CNI (tacrolimus or cyclosporine): 0.05–0.075 mg/kg/d tacrolimus given orally in two divided doses 12 h apart for 6–12 months without prednisone or 3.5–5.0 mg/kg/d cyclosporine given orally in two equally divided doses 12 h apart with 0.15 mg/kg/d prednisone for 6 months. Data are presented as the number of positive cases (% of total cases). Chi-square test was used to compare the three groups. *p* < .05 was shown in bold.

**Figure 2.**
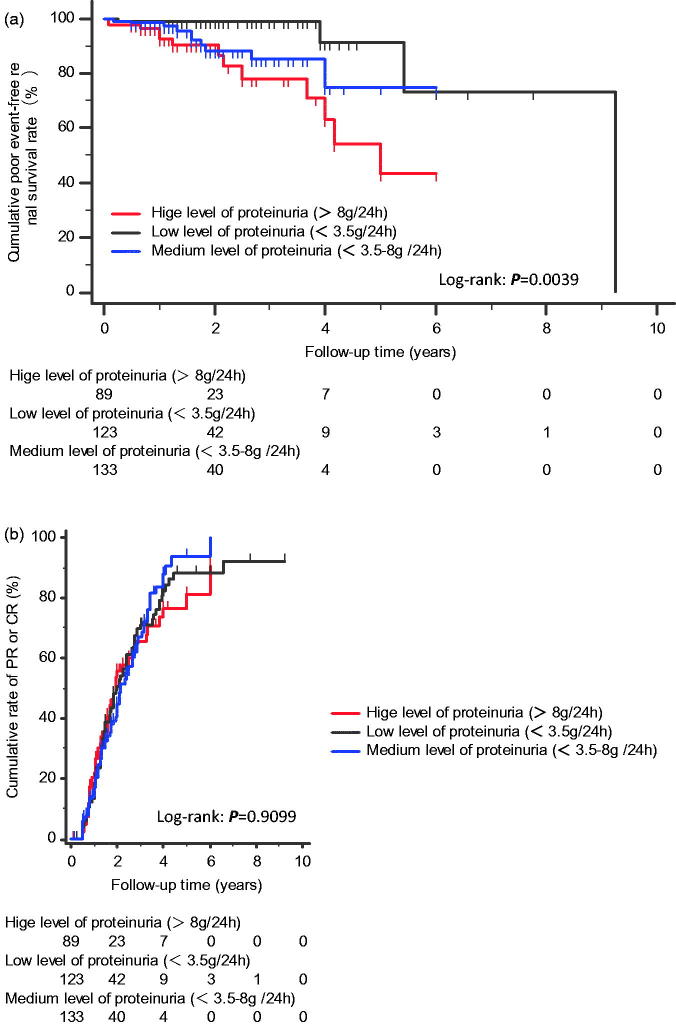
The Kaplan–Meier analysis. (a) Cumulative poor event-free renal survival rates in patients with low, medium, or high levels of proteinuria. (b) Cumulative incidence rates of PR or CR in patients with low, medium, or high levels of proteinuria.

The K–M analyses were used to compare the prognostic characteristics of the three groups to determine the cumulative poor event-free renal survival and cumulative incidence rates of PR or CR. The K–M analyses included patients with more than 6 months of follow-up, with 123 patients in the low group, 133 patients in the medium group and 89 patients in the high group. Patients in the high group had a significantly lower cumulative poor event-free renal survival rate than those of patients in the low and medium groups (odds ratio: 0.1846 and 0.5975; 95% confidence interval (CI): 0.07150–0.4768 and 0.2199–1.6238, respectively; *p*= .0039). However, the cumulative incidence rates of PR or CR were comparable among the three groups (*p*= .9099) (Figure 2).

## Discussion

In China, the incidence of MN has been rapidly increasing among patients with primary glomerulopathy, from 7.1% in 2000 to 22.7% in 2009–2011 [17]. MN is twice as common in men as in women, with disease onset at 50 years or older (39.64%), and it is the most common primary glomerular disease in patients over 60 years of age. Pan et al. [17] found that the proportion of elderly patients with MN has significantly increased from 3.18% in 1997–1999 to 15.21% in 2009–2011 (*p*< .001). In our current study, male patients were more likely to have high levels of proteinuria than were female patients, and the blood pressure of patients increased with increasing proteinuria. Additionally, the proportion of patients with edema and microscopic hematuria increased with an increase in proteinuria, indicating that the severity of symptoms increased with proteinuria level. These conclusions were further supported by an increase in the levels of SCr, TP, ALB, and IgG and eGFR with increasing proteinuria. However, higher levels of C4 and fibrinogen in blood and higher C3 fluorescence and lower fibrinogen fluorescence in tissues indicated that the complement and coagulation systems were active in IMN.

Although pathological stages were comparable among the low, medium and high groups, the extent of segmental sclerosis and balloon adhesion and renal interstitial lesion stage increased in severity with increasing proteinuria, consistent with clinical symptoms, indicating a close association between clinical manifestations and pathological changes.

Furthermore, patients in the low group were more likely to choose RAS receptor inhibitors, while patients in the high group were more likely to choose GC + CTX or CNIs. These observations were consistent with the suggestion of the 2012 KDIGO guidelines [16], whereby IMN patients are considered for treatment with immunosuppressive agents in the presence of nephrotic syndrome and persistent proteinuria exceeding 4 g/24 h and remaining higher than 50% of the baseline value.

According to one study, persistent heavy proteinuria is the most reliable predictor of life-threatening complications and poor renal outcomes in IMN patients, and patients who had sub-nephrotic-range proteinuria during the observation period had better outcomes than patients who progressed from sub-nephrotic-range proteinuria to nephrotic-range proteinuria [18]. In our current study, patients with high levels of proteinuria had significantly lower cumulative poor event-free renal survival rates than patients with low and medium levels of proteinuria, indicating that the extent of proteinuria is indicative of poor prognosis. Additionally, irrespective of different treatments and even in the presence of treatment with GC or immunosuppressive agents, the prognosis of patients was associated with baseline proteinuria level at the time of renal biopsy; the greater the extent of proteinuria, the poorer was the prognosis. However, the cumulative incidence rates of PR or CR were comparable among the three groups, indicating that after more than 6 months of treatment, the difference in survival among the three groups of patients disappeared. Furthermore, proteinuria level in the high groups became close to the level in the low group, confirming that the treatment was effective. Nevertheless, differences still existed among the groups in terms of poor prognosis indicated by the occurrence of a 50% reduction in eGFR, ESRD, or all-cause mortality. Thus, baseline proteinuria level was indicative of prognosis.

We can conclude that patients with IMN with different levels of proteinuria have unique clinical and renal histopathological characteristics, and baseline proteinuria level is indicative of prognosis in IMN patients; the greater the extent of proteinuria is, the worse the prognosis.
